# Electronic health record evidence suggests undercoding, underrecognition, and low antifibrotic use in progressive pulmonary fibrosis

**DOI:** 10.1371/journal.pone.0351328

**Published:** 2026-08-28

**Authors:** Bradley A. Sheffield, Brian J. Cassel, Apostolos Perelas, Patricia Sime, Peter Jackson

**Affiliations:** 1 Virginia Commonwealth University School of Medicine, Richmond, Virginia, United States of America; 2 Division of Hematology, Oncology and Palliative Care, Virginia Commonwealth University, Richmond, Virginia, United States of America; 3 Division of Pulmonary and Critical Care Medicine, Virginia Commonwealth University, Richmond, Virginia, United States of America; Kurume University School of Medicine: Kurume Daigaku Igakubu Daigakuin Igaku Kenkyuka, JAPAN

## Abstract

**Background:**

Progressive pulmonary fibrosis (PPF) is a heterogeneous group of non-IPF ILDs characterized by fibrotic progression and poor prognosis. Antifibrotic therapies slow disease progression, yet widely variable real-world prevalence estimates suggest that PPF may be undercoded and potentially underdiagnosed. In preliminary TriNetX analyses using ICD-10 code J84.170, PPF prevalence was markedly lower than previous estimates. To explore a potentially undiagnosed and/or uncoded PPF cohort, we adapted a published PPF proxy algorithm and compared demographic, clinical, and treatment differences between formally coded patients and proxy-identified patients.

**Methods:**

We conducted a retrospective cohort study using the TriNetX U.S. Collaborative Network from 2021 to 2024. Coded PPF was defined by J84.170, excluding IPF. Three proxy PPF cohorts—sensitive, specific, and strict—were adapted from Olson et al. using the closest available TriNetX variables, combining non-IPF ILD diagnoses with progression criteria. Prevalence, antifibrotic use, demographics, and ILD subtype distributions were compared. Outcomes included respiratory failure, death, and lung transplant. Odds ratios (ORs) and hazard ratios (HRs) were calculated. Propensity matching included age, sex, race, and BMI; balance was assessed using standardized mean differences (SMD < 0.10).

**Results:**

Among 57,193,009 adults, coded PPF prevalence was 3.7 per 100,000 versus 250, 124, and 32 per 100,000 in the sensitive, specific, and strict proxy cohorts, respectively. Antifibrotic use was 18.3% in coded PPF, compared with 3.2% in the sensitive cohort (OR 0.15), 5.6% in the specific cohort (OR 0.34), and 8.6% in the strict cohort (OR 0.64). IPF treatment was higher than PPF treatment (28.5%; OR 1.97). Demographics and ILD subtype distributions were broadly similar, and clinical outcomes were comparable between coded and proxy cohorts. Relative to PPF, IPF had lower hazards of respiratory failure (HR 0.56) and death (HR 0.52), but higher transplant rates (HR 1.93).

**Conclusions:**

Proxy-defined cohorts identified clinically similar but largely untreated populations, supporting the possibility of an uncoded and/or unrecognized PPF cohort. EHR-based proxy algorithms may serve as scalable screening tools to identify high-risk patients for further clinical evaluation. These findings should be interpreted cautiously given the use of EHR-derived proxy markers and evolving awareness and coding practices for PPF over time. Further validation with chart review and integrated clinical data is needed.

## Introduction

Progressive pulmonary fibrosis (PPF) is a heterogeneous group of non-idiopathic pulmonary fibrosis (IPF) interstitial lung diseases (ILDs) with fibrotic progression and a poor prognosis similar to IPF [1,2]. Recent trials show antifibrotics like nintedanib and nerandomilast slow PPF-related lung function decline [3,4]. Real-world PPF prevalence estimates vary widely, from 6 to 290 per 100,000, likely reflecting diagnostic complexity, heterogeneous cohorts, and potential variability in recognition and coding practices [5–8]. In preliminary TriNetX analyses using ICD-10 J84.170, PPF prevalence was only 3.7 per 100,000.

**Fig 1:**
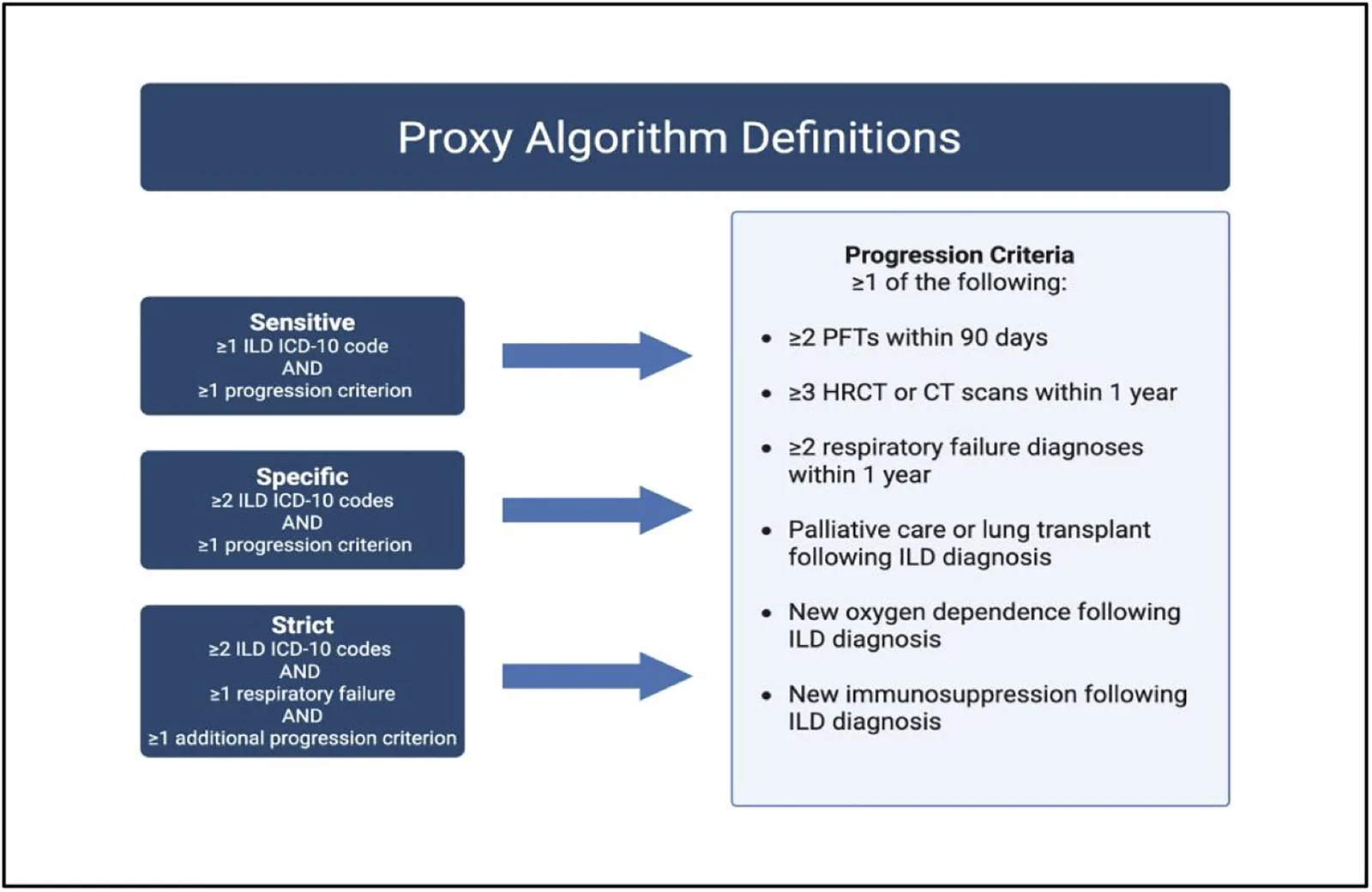
Progression criteria used to define proxy PPF cohorts, adapted from Olson et al. Each definition required an ILD ICD code and events suggesting disease progression, including repeat PFTs or HRCT/CT imaging, respiratory failure, palliative care, lung transplantation, oxygen dependence, or immunosuppression following ILD diagnosis. Abbreviations: interstitial lung disease (ILD); International Classification of Diseases (ICD); pulmonary function test (PFT); high-resolution computed tomography (HRCT); computed tomography (CT).

To explore a potentially underrecognized and uncoded cohort, we adapted a published PPF proxy algorithm [5] to construct three proxy cohorts in TriNetX (Fig 1). We compared coded and proxy cohort prevalence and antifibrotic use to quantify potential underrecognition, undercoding, and treatment differences. Although adjudication of proxy cohorts was not possible due to TriNetX data limitations, the much larger proxy-defined cohorts had similar mortality, demographics, and ILD subtypes to coded PPF, suggesting underrecognition, undercoding, and lower antifibrotic use. Because awareness and use of PPF coding evolved during the study period, differences between coded and proxy-defined cohorts may also reflect documentation practices as well as true differences in recognition.

## Materials and methods

We conducted a retrospective cohort study using the TriNetX U.S. Collaborative Network, including adults from 2021 to 2024 to capture the advent of the ICD-10 code for PPF. Coded PPF was defined using a single J84.170 code, excluding IPF. Proxy PPF cohorts were adapted from Olson et al. (2021) [5] using the closest available TriNetX equivalents and combined non-IPF ILD diagnoses with progression criteria in three variants (Fig 1 and S1 Appendix in S1 File). All queries were performed between June and September 2025. Cohorts were defined using prespecified rule-based criteria within the TriNetX platform.

We calculated prevalence and antifibrotic uptake and compared demographics, including age, sex, race, and ILD subtypes, with coded PPF. In a separate proxy agreement analysis, using J84.170 as a pragmatic diagnostic benchmark, we estimated proxy sensitivity, specificity, κ, Youden’s J, and prevalence-adjusted bias-adjusted κ (PABAK). Odds ratios (ORs) and hazard ratios (HRs) for antifibrotic uptake, lung transplant, respiratory failure, and death were calculated in TriNetX. We benchmarked coded PPF outcomes against IPF, the best-characterized fibrotic ILD with comparable outcomes [1,2]. Propensity matching used age, sex, race, and BMI. Group comparisons were summarized with standardized mean differences (SMDs). SMD < 0.10 indicated negligible differences.

## Results

Baseline characteristics are shown in Table 1.

**Table 1:** Baseline characteristics of patients with IPF and PPF cohorts defined by coded and proxy algorithms. For each cohort, we report sample size, prevalence per 100,000, antifibrotic use, mean age at diagnosis (± SD), and key demographic proportions, including sex, race, and presumptive ILD diagnoses. Standardized mean differences (SMDs) are reported for each proxy cohort versus the coded PPF cohort; values <0.10 were considered to indicate negligible imbalance. Abbreviations: SMD, standardized mean difference; CTD, connective tissue disease. CTD, rheumatoid arthritis, systemic sclerosis, myositis, and dermatomyositis all fall under systemic autoimmune rheumatic disease-associated interstitial lung disease (SARD-ILD).

Characteristic	IPF	Coded PPF (Ref)	Sensitive PPF	SMD	Specific PPF	SMD	Strict PPF	SMD
Total N	38,185	2,088	142,878	—	70,612	—	18,490	—
Prevalence (per 100k)	66.8	3.7	250.2	—	123.5	—	32.3	—
Antifibrotic Use, %	28.5%	18.3%	3.2%	—	5.6%	—	8.6%	—
Demographics								
Age, mean ± SD	69.4 ± 12.3	68.9 ± 13	67.7 ± 13.9	0.08	68.1 ± 13.5	0.05	69.8 ± 13.2	0.07
Sex, %								
*Female*	42%	54%	54%	0.01	55%	0.01	53%	0.03
*Male*	58%	46%	46%	0.01	45%	0.01	47%	0.03
Race, %								
*White*	78%	69%	73%	0.09	73%	0.08	74%	**0.12**
*Black*	9%	16%	15%	0.04	14%	0.06	13%	0.08
*Other*	3%	5%	4%	0.09	4%	0.07	4%	0.07
Presumptive ILD Subtype								
*CTD*	—	25%	15%	**0.26**	20%	**0.12**	21%	**0.11**
*Exposure*	—	18%	11%	**0.17**	11%	**0.14**	11%	**0.18**
*Rheumatoid Arthritis*	—	16%	11%	**0.14**	13%	0.09	12%	0.09
*Sarcoidosis*	—	5%	4%	0.03	4%	0.01	4%	0.03
*Systemic Sclerosis*	—	7%	4%	0.12	6%	0.04	6%	0.01
*Myositis*	—	7%	5%	0.06	6%	0.03	5%	0.06
*Dermato-* *myositis*	—	5%	2%	**0.14**	3%	0.1	3%	0.09
*Hypersensitivity pneumonitis*	—	7%	2%	**0.26**	3%	**0.19**	4%	**0.16**

Additional data underlying the results, figures, and tables are provided in S1–S4 Tables in S1 File.

### Prevalence

Among 57,193,009 adults, PPF prevalence was 3.7 per 100,000 for coded cases versus 250, 124, and 32 per 100,000 for the sensitive, specific, and strict proxy-defined cohorts, respectively.

### Treatment

Antifibrotics were prescribed to 18.3% of coded PPF patients, higher than all proxy cohorts: sensitive, 3.2% (OR 0.15, 95% CI 0.08–0.26); specific, 5.6% (OR 0.34, 95% CI 0.23–0.51); and strict, 8.6% (OR 0.64, 95% CI 0.46–0.90). IPF antifibrotic treatment was 28.5% (OR 1.97, 95% CI 1.49–2.62).

### Demographics

The specific proxy cohort was most similar to coded PPF: age, 68.1 versus 68.9 years; sex, 55% versus 54% female; and race, 73% versus 69% White and 14% versus 16% Black. All SMDs were <0.10 except White race in the strict proxy cohort (0.12).

### Subtype distribution

ILD subtype diagnoses were broadly similar between coded and proxy cohorts, with several diagnosis-level SMDs < 0.10. Larger imbalances included CTD-associated ILD and hypersensitivity pneumonitis in the sensitive proxy cohort, 15% versus 25% and 2% versus 7%, respectively, with SMDs of 0.26, which were small to moderate.

### Clinical outcomes

**Fig 2:**
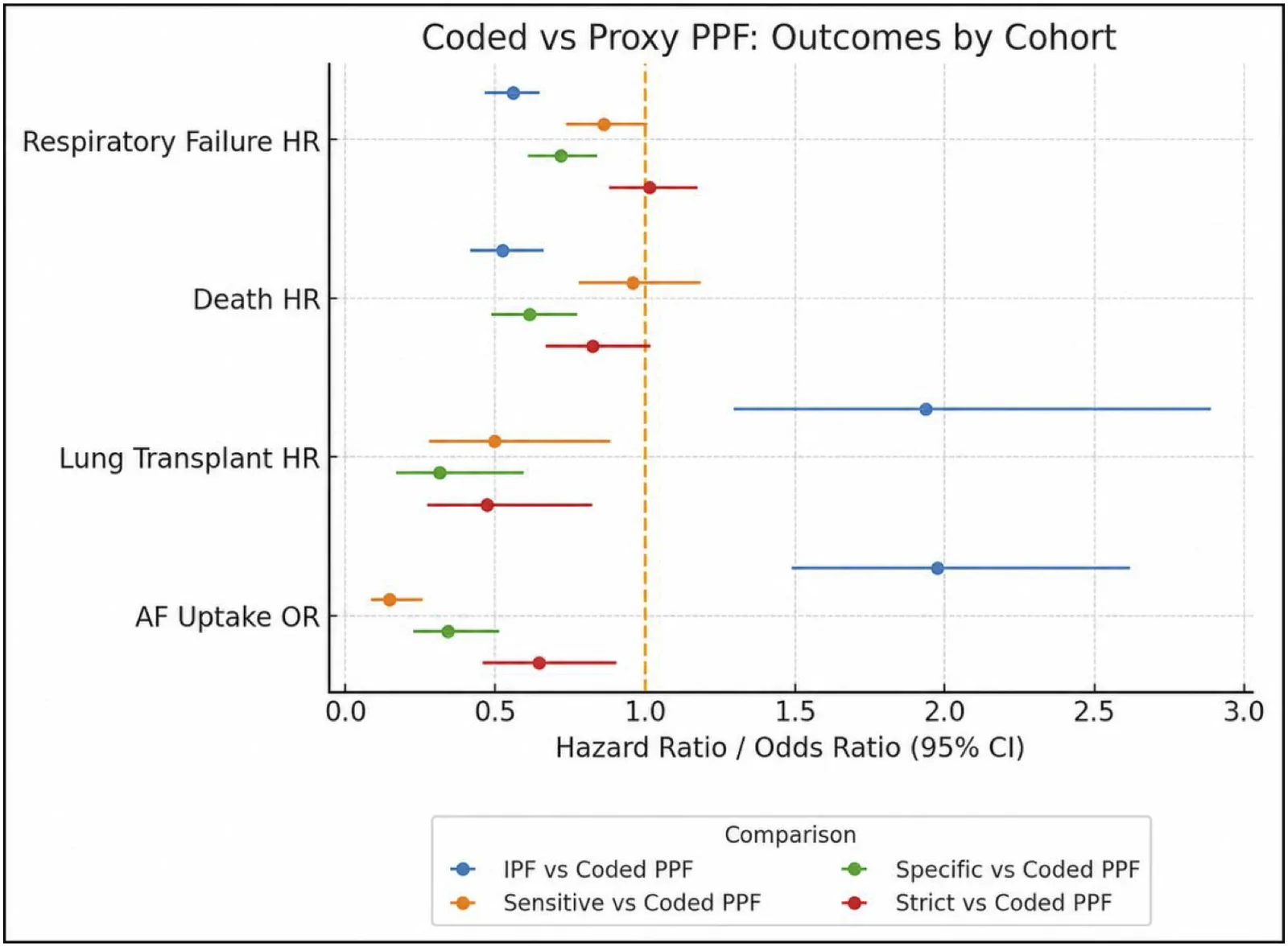
Forest plot of hazard ratios for respiratory failure, all-cause mortality, and lung transplant comparing IPF, coded PPF, and proxy PPF cohorts. Cohorts were propensity-matched on age, sex, race, BMI, and FVC% predicted when available. The benchmark/reference cohort for all analyses is coded PPF.

Compared with PPF, IPF had lower hazards of respiratory failure (HR 0.56, 95% CI 0.47–0.65) and death (HR 0.52, 95% CI 0.42–0.66). Lung transplant was more frequent in IPF (HR 1.93, 95% CI 1.30–2.89). Outcomes were broadly similar overall between coded and proxy cohorts. The sensitive proxy cohort showed comparable respiratory failure (HR 0.86, 95% CI 0.74–1.01) and death (HR 0.96, 95% CI 0.78–1.19), but fewer transplants (HR 0.50, 95% CI 0.28–0.88). The specific proxy cohort had lower respiratory failure (HR 0.72, 95% CI 0.61–0.84), death (HR 0.61, 95% CI 0.49–0.77), and transplant (HR 0.31, 95% CI 0.17–0.60). The strict proxy cohort showed comparable respiratory failure (HR 1.01, 95% CI 0.88–1.18), slightly lower mortality (HR 0.82, 95% CI 0.67–1.02), and fewer transplants (HR 0.47, 95% CI 0.27–0.82) (Fig 2).

### Overlap and proxy performance

Among coded PPF patients, only 20%–49% met proxy criteria. Using J84.170 as the benchmark, the proxies showed moderate specificity, ranging from 63% to 95%, and high negative predictive values of approximately 99%. Despite low κ values of 0.01–0.04, likely reflecting the rarity of coded PPF, PABAK values of 0.26–0.90 and Youden’s J values of 0.13–0.24 were higher.

## Discussion

In this large multicenter network, PPF proxy algorithms identified patients who were clinically, demographically, and etiologically similar to those with coded PPF, suggesting that PPF may be underrecognized and/or undercoded in real-world practice, with antifibrotic therapy concentrated among formally coded patients. Antifibrotic uptake in coded PPF was lower than in IPF (OR 1.97, 95% CI 1.49–2.62), consistent with recent Japanese data [9], and lower still in proxy cohorts. Coded patients had higher respiratory failure and mortality than proxy and IPF patients, suggesting the possibility that recognition and coding may occur after substantial progression and/or that the coded group represents a more aggressive phenotype. Additionally, it is likely that the PPF ICD-10 code is uncommonly used outside of pulmonology and is used to facilitate treatment, which may partly explain both its low use and higher treatment rates. Taken together, these data suggest two potential gaps in care: a substantial proportion of patients with possible PPF may go unrecognized or uncoded, and even among formally coded patients, antifibrotic treatment appears quite low. Importantly, these findings are suggestive rather than definitive, as proxy cohorts were defined using EHR-derived surrogate markers rather than confirmed clinical cases.

PPF is diagnostically challenging and may be delayed or missed, making EHR-based algorithms for case-finding appealing [10,11]. This challenge is reflected in the 2022 ATS/ERS/JRS/ALAT criteria, which defines PPF in patients with non-IPF ILD as at least two of three features occurring within the past year, without alternative explanation: worsening respiratory symptoms, physiological progression, or radiologic progression. Physiologic progression includes an absolute FVC decline of ≥ 5% predicted or an absolute DLCO decline ≥ 10% predicted within 1 year [12]. More consistent PPF coding may help improve EHR-based recognition, support clinical care and research, refine prevalence estimates, and clarify real-world antifibrotic treatment patterns.

Our proxies captured only approximately 20%–50% of coded PPF but demonstrated specificity of 63%–95% and NPV of approximately 99% for coded PPF, supporting their potential use as first-pass exclusionary screening tools. Despite low κ driven by coded PPF rarity, higher PABAK values of 0.26–0.90 and Youden’s J values of 0.13–0.24 support this strategy. The finding that only approximately 50% of the coded group met proxy criteria may reflect: (1) proxy-criteria fragmentation across centers that the federated TriNetX network cannot link, for example, one HRCT at three different hospitals does not equal three HRCTs; (2) progression data outside the TriNetX database, such as scanned PFTs, outside imaging, or notes; or (3) imperfect proxy criteria. Future adjudication studies embedding these proxies in EHRs with chart review may clarify these issues, allow iterative refinement of proxies, and may lead to earlier referral, timelier diagnosis, and earlier treatment for patients with PPF.

### Limitations

This study has several important limitations. First, because TriNetX provides aggregate, de-identified EHR data, we were unable to perform individual chart review or manual adjudication to confirm whether patients met formal PPF criteria. This is particularly important because PPF is defined longitudinally and requires clinical interpretation of symptoms, physiologic progression, and radiologic progression over time. Second, detailed PFT and CT data were unavailable in TriNetX, limiting the ability of the proxy definitions to capture true progression. As a result, the proxy cohorts should not be interpreted as confirmed PPF cases, but rather as rule-based EHR phenotypes intended to explore possible under-recognition and treatment patterns. Finally, coding practices, clinical awareness of PPF, and antifibrotic prescribing patterns may have evolved during the study period, which could influence comparisons between coded and proxy-defined cohorts. Future chart-adjudication studies are needed to validate these proxy definitions, refine EHR-based case-finding approaches, and determine how well these cohorts correspond to clinically confirmed PPF.

## Supporting information

S1 File Supplemental proxy definitions and data. This file contains Tables S1–S4.
(DOCX)
