# Supplementary material for: Electronic health record evidence suggests undercoding, underrecognition, and low antifibrotic use in progressive pulmonary fibrosis
Source: PLoS One. 2026 Aug 28;21(8):e0351328. doi: 10.1371/journal.pone.0351328 (PMC13524242; doi:10.1371/journal.pone.0351328)
Supplement: Supplementary file 1 [file pone.0351328.s001.docx]

**Supplemental Data**

**Summary Data Underlying Figures and Tables**

**Table S1. Cohort sizes, prevalence, and antifibrotic use underlying Table 1.**

| **Cohort** | **N (patients)** | **Population denominator** | **Prevalence (per 100,000)** | **Antifibrotic use** |
| --- | --- | --- | --- | --- |
| IPF | 38,185 | 57,193,009 | 66.8 | 10,883/38,185 (28.5%) |
| Coded PPF (J84.170) | 2,088 | 57,193,009 | 3.7 | 381/2,088 (18.3%) |
| Sensitive proxy PPF | 142,878 | 57,193,009 | 250 | 4,544/142,878 (3.2%) |
| Specific proxy PPF | 70,612 | 57,193,009 | 124 | 3,928/70,612 (5.6%) |
| Strict proxy PPF | 18,490 | 57,193,009 | 32 | 1,586/18,490 (8.6%) |

**Table S2. Odds ratios for antifibrotic use relative to coded PPF.**

| **Cohort** | **OR** | **95% CI** |
| --- | --- | --- |
| IPF | 1.97 | 1.49-2.62 |
| Sensitive proxy PPF | 0.15 | 0.08-0.26 |
| Specific proxy PPF | 0.34 | 0.23-0.51 |
| Strict proxy PPF | 0.64 | 0.46-0.90 |

**Table S3. Hazard ratios for clinical outcomes relative to coded PPF.**

| **Cohort** | **Outcome** | **HR** | **95% CI** |
| --- | --- | --- | --- |
| IPF | Respiratory failure | 0.56 | 0.47-0.65 |
| IPF | Death | 0.52 | 0.42-0.66 |
| IPF | Lung transplant | 1.93 | 1.30-2.89 |
| Sensitive proxy PPF | Respiratory failure | 0.86 | 0.74-1.01 |
| Sensitive proxy PPF | Death | 0.96 | 0.78-1.19 |
| Sensitive proxy PPF | Lung transplant | 0.50 | 0.28-0.88 |
| Specific proxy PPF | Respiratory failure | 0.72 | 0.61-0.84 |
| Specific proxy PPF | Death | 0.61 | 0.49-0.77 |
| Specific proxy PPF | Lung transplant | 0.31 | 0.17-0.60 |
| Strict proxy PPF | Respiratory failure | 1.01 | 0.88-1.18 |
| Strict proxy PPF | Death | 0.82 | 0.67-1.02 |
| Strict proxy PPF | Lung transplant | 0.47 | 0.27-0.82 |

**Table S4. Proxy agreement and performance against coded PPF (J84.170) as reference.**

| **Proxy cohort** | **TP** | **FP** | **FN** | **TN** | **Sensitivity (%)** | **Specificity (%)** | **PPV (%)** | **NPV (%)** |
| --- | --- | --- | --- | --- | --- | --- | --- | --- |
| Sensitive | 1,380 | 135,910 | 1,413 | 233,822 | 49.4 | 63.2 | 1.0 | 99.4 |
| Specific | 1,167 | 67,115 | 1,626 | 302,617 | 41.8 | 81.9 | 1.71 | 99.5 |
| Strict | 568 | 17,200 | 2,225 | 352,532 | 20.3 | 95.4 | 3.2 | 99.4 |

**Note.** Table S4 reflects a separate exploratory proxy agreement/performance query performed in TriNetX on a different query date than the final prevalence estimates shown in Table S1. Because TriNetX is a live federated EHR network, cohort counts may vary across query dates due to data refreshes, site participation, and record updates. Therefore, Table S4 should be interpreted as an agreement/performance analysis rather than as the denominator for prevalence estimation.
